# Antioxidant Nanotherapies for Intervertebral Disk Degeneration: Progress and Prospects

**DOI:** 10.3390/antiox15060745

**Published:** 2026-06-11

**Authors:** Yingzi Zhou, Yihang Fan, Yuxuan Hu, Huihui Wang

**Affiliations:** 1 The First School of Clinical Medicine, Faculty of Medicine, Yangzhou University, Yangzhou 225009, China; ascend_med@163.com (Y.Z.); 19805131811@163.com (Y.F.); hu1xion@163.com (Y.H.); 2 School of Traditional Chinese Medicine, Faculty of Medicine, Yangzhou University, Yangzhou 225009, China; 3 The Key Laboratory of the Jiangsu Higher Education Institutions for Nucleic Acid & Cell Fate Regulation (Yangzhou University), Yangzhou 225001, China

**Keywords:** intervertebral disk degeneration, nanomaterials, nanozymes, oxidative stress, cell senescence, drug delivery

## Abstract

Intervertebral disk degeneration (IVDD) is widely recognized as a major contributor to discogenic low back pain (LBP), imposing a substantial burden on global public health and socioeconomic systems. Growing evidence confirms that disrupted redox homeostasis, excessive reactive oxygen species (ROS) accumulation, and oxidative stress act as major convergent mechanisms that propagate inflammatory cascades, nucleus pulposus cell dysfunction, and extracellular matrix degradation. Although conventional conservative therapies and surgical interventions are clinically effective in relieving macrostructural compression, they remain limited in resolving localized molecular dysregulation. In recent years, nanotechnology has emerged as a promising strategy for overcoming the limitations of traditional therapy for IVDD. This review provides an analysis of four categories of antioxidant nanotherapies for IVDD, including inorganic functional nanozymes, bioactive nanomaterials, stimuli-responsive nanosystems, and nanocomposite scaffolds. We elaborate on their mechanisms in scavenging excessive ROS, restoring redox equilibrium, protecting mitochondrial function, and ameliorating oxidative stress-induced degeneration. Integrating structural biomimicry with microenvironmental responsiveness enables the engineering of composite nanosystems with multi-pathway ROS-scavenging capabilities. Therefore, these platforms emerge as promising therapeutic strategies for arresting IVDD progression. Finally, we discuss the key obstacles to clinical translation. Overall, this review provides insights into the development of redox-targeted therapies.

## 1. Introduction

Low back pain (LBP) is among the leading causes of disability and poor productivity worldwide. Epidemiological data from 1990 to 2019 reveal that LBP consistently remained the leading cause of disability, inflicting not only physical suffering on patients but also imposing a substantial societal burden [1]. Disk degeneration is highly prevalent in asymptomatic individuals, and not all cases of LBP in degenerative changes are discogenic in origin [2]. Intervertebral disk degeneration (IVDD) is a major cause of discogenic LBP, accounting for approximately 40% of cases of pain due to disc pathology [3]. Therefore, the significant epidemiological impact and social burden of IVDD necessitate the development of more effective and durable treatment strategies.

IVDD is a complex process involving mechanical and biological components. However, the “degenerative cycle” theory proposed by Vergroesen et al. provides a concise and simplified description of this pathological process [4]. In their theoretical model, abnormal mechanical loading, cellular metabolic dysfunction, and extracellular matrix (ECM) degradation are integrated into a vicious cycle wherein any component can serve as an “entry point” to initiate disease progression. This process is continuously amplified, ultimately leading to the destruction of intervertebral disk (IVD) structural integrity and loss of biomechanical function. Importantly, although IVDD is initiated by a multifactorial interplay of mechanical loading, aging, genetics, and metabolic stress, growing evidence has identified disrupted redox homeostasis and excessive reactive oxygen species (ROS) accumulation as critical convergent mechanisms through which these diverse upstream stressors intersect to amplify this vicious cycle [5,6].

As an avascular tissue, the IVD relies primarily on diffusion from capillaries within the cartilaginous endplates for oxygen and nutrient supply [5]. Consequently, its intrinsic self-repair capacity is extremely limited under normal conditions. Once damage occurs and IVDD develops, the energy supply becomes further restricted, and the disk enters a persistent state of hypoxia and nutrient deprivation [7,8]. These factors disrupt the balance between oxidative and antioxidant systems within the IVD, leading to excessive ROS production, which in turn aggravates pathological changes such as cellular dysfunction, inflammation, and matrix degradation [6,9,10].

These complex microenvironmental alterations collectively contribute to the difficulty of fully recovering from IVDD and represent a formidable barrier that novel therapeutic strategies aim to address. Consequently, restoring redox homeostasis within the IVD is increasingly recognized as a pivotal component of effective therapeutic intervention. Based on this pathological demand, nanomaterials possessing potent antioxidant and precise targeting capabilities offer a promising avenue for stabilizing the microenvironment and delaying IVDD progression.

Current clinical management of IVDD primarily addresses symptomatic discomfort and mechanical stabilization rather than holistic biological restoration. Conservative options, including pharmacological and physical therapies, provide indispensable short-term symptom control but are limited in their ability to arrest ongoing tissue degeneration. Mechanistically, systemically administered drugs cannot effectively penetrate the avascular and dense IVD barrier to reach effective therapeutic concentrations in the nucleus pulposus [11]. Long-term use of nonsteroidal anti-inflammatory drugs carries risks of gastrointestinal, cardiovascular, and renal toxicity, especially in elderly patients [12]. Local injection therapies lack controlled-release carriers, leading to rapid drug clearance and insufficient sustained effects [13]. Similarly, major surgical interventions such as spinal fusion and total disc replacement are highly effective in relieving severe neural compression and restoring spinal alignment. Nevertheless, they frequently alter natural spinal biomechanics, which may sacrifice segmental mobility, increase the risk of adjacent segment degeneration, or predispose patients to prosthesis-related complications [11,14,15]. Consequently, novel biological therapeutic strategies capable of overcoming the anatomical barriers of the IVD, enabling precise drug delivery, and exerting antioxidant effects are urgently needed.

Regenerative medicine approaches have emerged to address these limitations but are hindered by poor cell survival, rapid payload degradation, and low targeting efficiency in the oxidative and hostile IVD microenvironment [16,17]. Nanomaterials provide a transformative solution by virtue of their nanoscale size, high surface area, tunable targeting, and functional versatility. They can serve as effective carriers for the aforementioned regenerative factors, protecting them from rapid clearance within the body. In particular, nanomaterials enable precise regulation of oxidative stress, direct ROS scavenging via antioxidant nanozymes, on-demand drug release through redox-responsive systems, and restoration of redox signaling.

Distinct from existing reviews that broadly summarize the applications of nanomaterials for IVD regeneration, this review uniquely centers on oxidative stress, ROS regulation, redox signaling, antioxidant nanozymes, and redox-responsive therapeutic systems—the redox-centric mechanisms that underlie IVDD progression and treatment. While recent reviews have focused on general regenerative nanomaterials, this study systematically consolidates advances in redox-targeted nanotherapies, clarifies their mechanisms in restoring IVD redox homeostasis, and evaluates translational challenges. This focused perspective fills a critical gap in the field and provides a specialized reference for developing antioxidant-based nanotherapies for IVDD.

## 2. Literature Search Methodology

### 2.1. Search Strategy and Data Sources

To ensure a comprehensive and reproducible collection of relevant literature, a systematic search was conducted across two electronic databases, PubMed and Web of Science, covering publications from January 2015 to May 2026. The search strategy combined Medical Subject Headings (MeSH) terms and free-text keywords using Boolean operators. The primary search string was structured as follows: (“intervertebral disk degeneration” OR “IVDD” OR “nucleus pulposus”) AND (“nanomaterials” OR “nanozymes” OR “nanoparticles” OR “exosomes” OR “extracellular vesicles” OR “liposomes” OR “polymeric micelles” OR “ROS-responsive” OR “stimuli-responsive” OR “hydrogel”) AND (“oxidative stress” OR “reactive oxygen species” OR “redox balance”).

Recognizing that highly influential mechanistic or cross-disciplinary studies may not be captured by a narrowly defined search strategy, the database search was supplemented by manual screening and backward and forward citation tracking of key articles. This supplementary approach was used to identify: (1) foundational epidemiological and molecular studies on disc degeneration independent of material-based interventions; and (2) relevant biomaterial studies originally validated in related musculoskeletal disorders, particularly osteoarthritis and joint cartilage degeneration, that may provide translational insights relevant to the IVDD microenvironment.

### 2.2. Inclusion and Exclusion Criteria

Studies were selected according to pre-defined eligibility criteria. Inclusion criteria comprised: (1) original research evaluating the antioxidant, anti-inflammatory, or regenerative effects of nanomaterials in IVDD pathogenesis; (2) in vitro studies using disk-derived cells (e.g., nucleus pulposus cells or endplate chondrocytes); and (3) in vivo studies employing established animal models of IVDD or related musculoskeletal disorders when they provide mechanistic insights relevant to IVDD pathophysiology. Studies investigating the catalytic mechanisms of inorganic functional nanozymes or the long-term biosafety of nanomaterials were also included, even when conducted outside of explicit IVDD models, as these studies provide important information for potential clinical translation. Exclusion criteria included non-English publications, editorials, case reports, and studies lacking characterization of nanomaterial platforms.

### 2.3. Screening Strategy

Literature screening followed a standardized two-stage process. A total of 163 records were initially retrieved from the electronic databases. Two investigators independently screened the titles and abstracts, resulting in the exclusion of 34 duplicate or irrelevant records. Subsequently, full-text assessments were conducted for the remaining 129 articles. To enhance the mechanistic and translational scope of the review, an additional 53 publications, including foundational studies on disk pathobiology, animal validation models, and relevant nanomaterial research from related musculoskeletal disorders, were identified through manual screening and backward and forward citation tracking. Following a comprehensive quality assessment of the combined literature pool, 111 publications were ultimately included in this review.

## 3. IVDD: From Physiology to Pathology

### 3.1. Normal Structure and Function of the IVD

**Figure 1:**
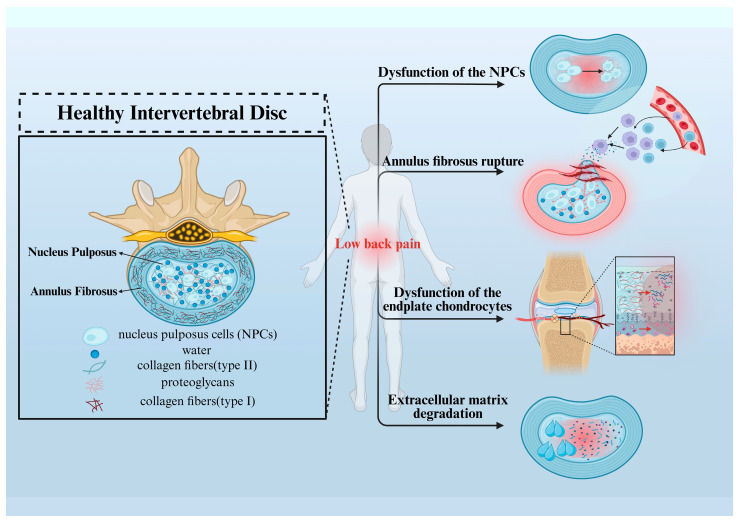
Normal structure of the intervertebral disk and manifestations of structural damage in intervertebral disk degeneration. (The red arrow indicates the transition from normal to degenerated structure.) Created in BioRender. Yingzi Zhou. (2026). https://BioRender.com/bj7rzy1 (accessed on 21 April 2026).

The IVD is a fibrocartilaginous structure composed of the nucleus pulposus (NP), annulus fibrosus (AF), and cartilaginous endplate (CEP). Located between adjacent vertebral bodies, the IVD bears gravitational loads, absorbs shock and mechanical stress, and assists the spine in performing movements such as flexion and rotation, thereby maintaining spinal flexibility [18,19]. Figure 1 provides a conceptual overview of the normal IVD structure and primary pathological manifestations of IVDD discussed in later sections.

#### 3.1.1. NP

The NP is a gel-like tissue located at the center of the IVD and is characterized by high water content and excellent elasticity [20]. The NP is primarily composed of nucleus pulposus cells (NPCs) and the extracellular ECM in which they reside. Proteoglycans and type II collagen fibers are the key components responsible for the physiological functions of the ECM [19], particularly the sulfated glycosaminoglycans within proteoglycans. Their negatively charged side chains retain water, and this highly hydrated state enables the NP to withstand compressive forces, thereby providing the functional basis for cushioning spinal pressure [20,21]. The early stages of IVDD are often characterized by NPC degeneration, primarily manifested as loss of NPCs, reduced ECM hydration, and alterations in matrix composition, all of which lead to a decline in the cushioning and load-bearing capacity of the NP [18].

#### 3.1.2. AF

The AF is a concentric ring-shaped structure surrounding the NP and comprises multiple layers of dense lamellae. Each lamella is rich in type I and type II collagen fibers, which are arranged obliquely relative to the axis of compression, forming a robust ring-shaped structure with high tensile strength and toughness [21]. The layered organization of the AF not only effectively prevents NP herniation and subsequent nerve root compression during mechanical loading, thereby reducing pain [19], but also works in concert with the CEP to form a natural physical barrier that separates the components of the NP from the body’s immune system, thereby establishing an “immune-privileged zone” [17].

When the AF is damaged and NP protrusion occurs, substances that normally remain confined within the NP region are released. These substances may then be recognized as “foreign bodies” by the immune system, triggering inflammation and autoimmune reactions [21]. Furthermore, the AF is instrumental in maintaining the structural stability of the IVD, limiting excessive vertebral movement, and withstanding mechanical stress.

#### 3.1.3. CEP

The CEP is located between the IVD and the superior and inferior vertebral bodies. It is a thin layer of hyaline cartilage composed primarily of type II collagen fibers and proteoglycans. Since the IVD is an avascular tissue, the capillaries within the CEP serve as the primary pathway for nutrient diffusion, including glucose and oxygen, as well as for metabolic waste clearance between the vertebral vasculature and IVD [20,21]. However, during IVD degeneration, the CEP undergoes calcification and erosion, which significantly impair nutrient diffusion, thereby exacerbating cellular nutrient deficiency and IVDD [20].

### 3.2. Key Pathogenic Mechanisms of IVDD

**Figure 2:**
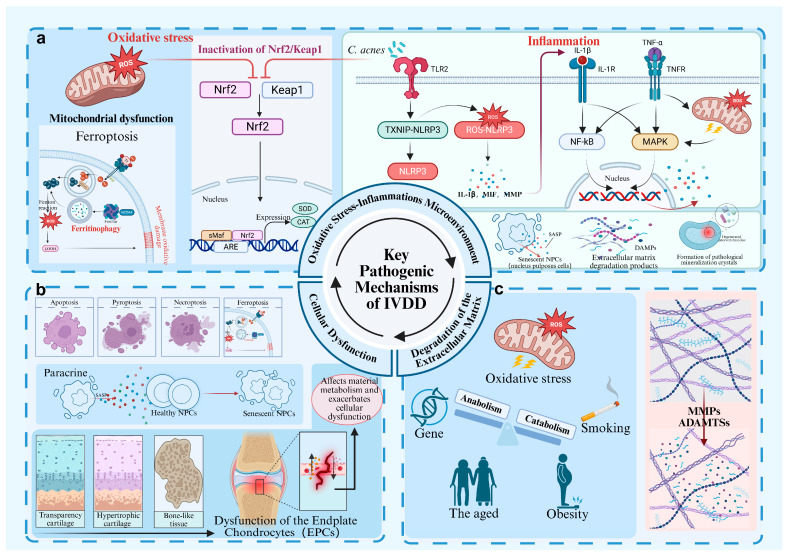
Key pathogenic mechanisms of intervertebral disk degeneration. (**a**) Mitochondrial dysfunction and inflammatory responses lead to Nrf2/Keap1 dysregulation. (**b**) Dysfunction of nucleus pulposus cells (NPCs) and endplate chondrocytes (EPCs). (**c**) The extracellular matrix (ECM) undergoes degradation under the action of various catabolic enzymes. Created in BioRender. Yingzi Zhou. (2026). https://BioRender.com/qwiv7qk (accessed on 13 May 2026).

IVDD is a complex process driven by multiple environmental and intrinsic factors. Abnormal mechanical loading [4], external trauma [4,22], unhealthy lifestyle habits [23], and genetic factors [24] can contribute to IVDD development. Through a series of complex pathophysiological processes, these factors interact synergistically, ultimately leading to dysfunction of cellular biological functions within the IVD [22,23]. However, the core pathological mechanisms underlying IVDD progression ultimately involve three key processes: the formation of an inflammatory and oxidative stress microenvironment, cellular dysfunction, and ECM degradation, as schematically illustrated in Figure 2. These processes are causally interconnected and mutually reinforcing, creating a vicious cycle that not only initiates IVDD but also accelerates disease progression. Crucially, redox imbalance serves as a pivotal convergent hub and a major driving force that propagates this vicious cycle.

#### 3.2.1. Redox Imbalance and Oxidative Stress: A Major Convergent Hub in IVDD

Oxidative stress refers to a pathological state of redox imbalance caused by excessive ROS production and insufficient antioxidant defense capacity [25]. It is increasingly recognized as a central pathological crossroad in the development of IVDD. As the largest avascular tissue in the human body, the healthy IVD exists within a highly specialized microenvironment. Both endogenous and exogenous stimuli can disrupt redox balance, leading to excessive ROS accumulation that exceeds the cellular clearance capacity, thereby accelerating oxidative stress cascades [5]. This disruption of redox homeostasis not only exacerbates cellular damage but also participates in a self-amplifying feedback loop with localized inflammatory responses. Together, these interconnected microenvironmental alterations are heavily implicated in driving cellular dysfunction and matrix catabolism, progressively propelling IVDD toward advanced degenerative stages.

##### Mitochondrial Dysfunction: The Cause of Excessive ROS Production

Mitochondrial dysfunction is widely recognized as a major intracellular source of excessive ROS production within the degenerating IVD microenvironment. Current experimental models suggest that stimulation by abnormal mechanical loading, as well as the presence of pro-inflammatory factors such as IL-1β and TNF-α, can accelerate abnormal mitochondrial fission and membrane potential depolarization in NPCs. These alterations disrupt the stability of the electron transport chain, subsequently contributing to mitochondrial dysfunction [26]. Damaged mitochondria generate large amounts of ROS during redox metabolism, at levels far exceeding the maximum clearance capacity of the cellular antioxidant defense system [27]. The excessive accumulation of ROS further exacerbates mitochondrial oxidative damage, thereby establishing a self-amplifying feedback loop in which mitochondrial impairment and unchecked ROS generation continuously fuel each other. Concurrently, excessive ROS accumulation is also associated with oxidative damage to cellular structures through DNA strand oxidation, protein oxidative modification, and lipid peroxidation—pathological events that are closely linked to impaired cell proliferation and accelerated transition toward a catabolic phenotype [28].

##### Dysregulation of the Nrf2/Keap1 Signaling Axis and Antioxidant Enzymes

The Nrf2/Keap1 signaling pathway constitutes a central defense axis for IVD cells against oxidative stress and maintenance of redox homeostasis. Under basal physiological conditions, Nrf2 remains sequestered by its negative regulator, Keap1, which actively targets Nrf2 for constitutive ubiquitination and subsequent 26S proteasomal degradation [29,30,31]. Upon elevation of intracellular ROS levels, conformational modifications within Keap1 disrupt this repressive complex, effectively blocking Nrf2 ubiquitination and liberating it for nuclear translocation. Once inside the nucleus, Nrf2 binds with small musculoaponeurotic fibrosarcoma (Maf) proteins to form a functional heterodimer. This Nrf2–Maf complex subsequently recognizes and binds to specific antioxidant response elements within genomic DNA, systematically mobilizing the transcription of downstream antioxidant enzymes, principally superoxide dismutase (SOD) and catalase (CAT), to neutralize pathologically accumulated ROS [25].

Tang et al. evaluated Nrf2 expression in NP tissue samples from patients with degenerative IVDs and found that the severity of IVD degeneration was negatively correlated with Nrf2 levels [30,31]. Reduced Nrf2 not only disrupts the Nrf2/Keap1 signaling axis and inhibits Nrf2 nuclear translocation, but also likely decreases the expression and activity of antioxidant enzymes such as SOD and CAT, thereby weakening the ROS scavenging capacity of cells within the IVD. Concurrently, this progressive collapse of the antioxidant defense system prevents effective containment of oxidative stress, further amplifying oxidative damage and serving as a key intrinsic cause of persistent redox imbalance and progressive IVDD development.

#### 3.2.2. Oxidative Stress and Inflammatory Responses

Within the degenerating IVD microenvironment, oxidative stress and inflammatory responses are highly coupled and mutually inducible [6,32], forming a positive feedback loop of “inflammation–mitochondrial damage–ROS–exacerbated inflammation” that accelerates IVDD progression. The inflammatory microenvironment within the IVD arises through the combined action of endogenous and exogenous factors.

Regarding endogenous factors, the senescence-associated secretory phenotype (SASP) secreted by senescent cells and damage-associated molecular patterns (DAMPs), such as fibronectin and hyaluronan fragments generated by ECM degradation, promote the release of pro-inflammatory cytokines including IL-6, IL-1β, and TNF-α via TLR2/TLR4 signaling [33]. Among exogenous factors, abnormal mechanical loading upregulates the mechanosensitive ion channel Piezo1, thereby promoting the production of pro-inflammatory mediators [22]. Furthermore, annular tears caused by abnormal mechanical loading and external trauma result in the loss of NP immune privilege, triggering autoimmune responses [34]. In addition, infection with *Cutibacterium acnes* (*C. acnes*) has been shown in specific models to induce pyroptosis via the TXNIP-NLRP3 and ROS-NLRP3 axes, releasing large amounts of inflammatory mediators and matrix-degrading enzymes [35].

Under persistent pathological conditions, these endogenous and exogenous factors collectively predispose the IVD microenvironment to sustained accumulation of pro-inflammatory cytokines, particularly IL-1β and TNF-α. These cytokines not only activate the NF-κB/MAPK inflammatory pathway, significantly enhancing the expression and activity of MMP-1, MMP-3, MMP-13, ADAMTS-4, and ADAMTS-5 and disrupting the catabolic–anabolic balance of NPCs, but also induce mitochondrial dysfunction [36]. More importantly, ROS function as intracellular secondary messengers within this inflammatory milieu. Mechanistic evidence derived from secondary spinal cord injury models [37] indicates that excessive ROS can directly activate MAPK signaling and subsequently activate the NF-κB pathway, thereby substantially amplifying inflammatory responses. Concurrently, progressive attenuation or conditional inactivation of the Nrf2/Keap1 antioxidant pathway in advanced IVDD fails to counteract excessive MAPK/NF-κB activation, further preventing termination of oxidative stress and inflammatory responses.

Linked by ROS, oxidative stress and inflammatory responses amplify each other, forming a pathological microenvironment that cannot spontaneously reverse. These findings highlight that although destructive signaling may be triggered by diverse initial insults, ranging from mechanical overload via Piezo1 activation to bacterial infection, their downstream pathological trajectories consistently converge on disrupted redox homeostasis. Therefore, oxidative stress is not merely an isolated initiator but rather a critical intermediary that translates diverse etiological stresses into progressive tissue degeneration.

#### 3.2.3. Oxidative Stress-Mediated Cellular Dysfunction and ECM Degradation

Persistent oxidative stress and prolonged stimulation by the inflammatory microenvironment further lead to cellular dysfunction and ECM degradation.

##### Oxidative Stress-Mediated Cellular Dysfunction

Cellular dysfunction is primarily characterized by the accumulation of cellular senescence and programmed cell death. Aging, abnormal mechanical loading, and prolonged stimulation by the inflammatory-oxidative stress microenvironment induce NPCs to enter a state of irreversible cell cycle arrest known as cellular senescence [38,39], in which oxidative stress serves as a major senescence-inducing stimulus [40,41]. Senescent NPCs remain metabolically active and continue to secrete SASP factors, thereby propagating senescence to neighboring healthy cells through paracrine mechanisms and rapidly expanding the degenerative region [42].

In addition to senescence, oxidative stress also mediates various forms of programmed cell death, among which ferroptosis is considered an important contributor to cellular degeneration [43]. Taking endplate chondrocytes (EPCs) as an example, specific in vitro and in vivo models indicate that severe oxidative stress can activate the YAP/TEAD1/NCOA4 axis within EPCs, initiating ferritinophagy and the release of free iron. Simultaneously, degenerating EPCs upregulate transferrin receptor 1 (TfR1) via HIF-2α, thereby contributing to iron-dependent lipid peroxidation and conditionally induce ferroptosis [44,45]. Ferroptosis is proposed not only to compromise EPC survival but also to promote their transformation into a hypertrophic phenotype, potentially accelerating pathological CEP calcification and impairing nutrient supply to the IVD [46].

Although direct evidence from IVDD models remains limited, insights from cartilage injury models suggest that the loss of structural homeostatic factors, particularly osteopontin, triggers aberrant activation of the NF-κB pathway [47]. Given the phenotypic and functional similarities between joint chondrocytes and EPCs, depletion of osteopontin may likewise accelerate EPC degeneration. Programmed cell death processes such as pyroptosis, necroptosis, and mitochondria-mediated apoptosis also occur under the influence of oxidative stress and inflammation. These forms of cell death are accompanied by cell membrane rupture and release of DAMPs, further exacerbating the degenerative microenvironment and creating a vicious cycle of “cell death–inflammation” [26,27].

##### Oxidative Stress-Mediated ECM Degradation

ECM degradation is a direct hallmark of structural deterioration in IVDD and is characterized by extensive degradation of aggrecan and type II collagen. The oxidative stress–inflammation axis drives metabolic imbalance within the ECM [48]. In healthy IVD, ECM synthesis and degradation remain in equilibrium. However, factors such as aging, abnormal mechanical loading, and smoking, mediated through oxidative stress, promote the transition of NPCs toward a catabolic phenotype [23,49].

Oxidative stress and inflammatory signaling significantly upregulate the expression of matrix metalloproteinases (MMPs) and a disintegrin and metalloproteinases with thrombospondin motifs (ADAMTSs) though activation of the NF-κB/MAPK pathway. Among these enzymes, MMP-3 and MMP-13 predominantly mediate collagen and proteoglycan degradation, whereas ADAMTS-4 and ADAMTS-5 specifically target aggrecan degradation [50]. Extensive ECM degradation leads to reduced NP hydration, decreased disk height, and loss of biomechanical function [49]. Concurrently, hyaluronan and fibronectin fragments generated during ECM degradation act as DAMPs that reactivate TLR signaling, thereby further amplifying inflammatory and oxidative stress responses and establishing a positive feedback loop between matrix degradation and inflammation that continuously drives IVDD progression.

Consequently, inflammation, oxidative stress, cellular dysfunction, and ECM degradation interact in a time-dependent and self-reinforcing cascade throughout IVDD progression. During the early stage, heterogenous insults, such as abnormal mechanical loading, microvascular impairment, and genetic susceptibility, alter the IVD microenvironment. These diverse stressors rapidly induce secondary redox imbalance and low-level ROS accumulation, which collective establish a pro-degenerative state and trigger mild inflammatory responses. This subtle disturbance causes early metabolic dysfunction in NPCs and modest impairment of ECM anabolism, with ECM degradation slightly exceeding synthesis at a potentially reversible stage.

During the intermediate stage, unresolved oxidative stress drives excessive ROS accumulation and amplifies inflammatory signaling, forming a positive feedback loop between oxidative stress and inflammation. This loop accelerates NPC senescence, apoptosis, and phenotypic transition toward catabolism, while ECM degradation becomes prominent and is accompanied by AF microdamage and incipient pathological neurovascular ingrowth.

In the advanced stage, chronic severe oxidative stress and sustained inflammatory stimulation lead to massive NPC loss, impaired matrix synthesis, and unrestrained ECM catabolism. Disk structural integrity is progressively destroyed, accompanied by marked ECM depletion, fissure formation, and extensive neurovascular invasion, ultimately resulting in irreversible disk collapse, biomechanical failure, and clinical discogenic pain. At this stage, the pathological cascade can no longer spontaneously reverse.

Given the complex pathological mechanisms underlying IVDD described above, traditional single-target inhibitors struggle to achieve effective repair. Consequently, the development of nanomaterials capable of multi-pathway ROS scavenging and mitochondrial targeting has emerged as a key research direction for alleviating redox imbalance, suppressing oxidative stress responses, and restoring IVD homeostasis.

## 4. Nanotherapeutic Strategies Targeting Redox Homeostasis in the IVD

Imbalances in redox homeostasis represent one of the major pathological drivers of IVDD progression. They not only exacerbate inflammatory responses but also aggravate a series of cascading reactions that lead to cellular dysfunction and ECM degradation. Compared with traditional therapeutic drugs or regenerative medical technologies, nanomaterials can achieve controlled drug release and tissue-targeted effects. To a certain extent, nanomaterials hold significant potential to mitigate the problems faced by the aforementioned treatment, such as the short half-life and the rapid clearance rate within the lesion. Meanwhile, conventional therapeutic agents struggle to penetrate the avascular physiological barrier of the IVD and are largely insufficient to comprehensively mitigate excessive ROS accumulation and oxidative stress-induced damage [51]. Owing to the rapid advancement of nanotechnology, the emergence of nanomaterials has provided novel approaches for addressing these challenges. The current development of nanomaterials for promoting functional recovery of the IVD under IVDD conditions is no longer limited to a single function. Instead, current integrated designs focus on three core functions—structural biomimicry [52], targeted delivery [11,53], and intrinsic biological activity [54]—enabling these nanomaterials to serve as a multifunctional platform for targeted regulation of IVD redox homeostasis, thereby potentially disrupting degenerative cascades.

First, nanocomposite scaffolds, such as nanocomposite hydrogels that provide structural support and biomimetic functions, can mimic the structure and mechanical properties of the natural ECM. In this way, they provide immediate structural support and a stable biomechanical environment within the IVD, thereby creating a supportive microenvironment conducive to cell survival and tissue regeneration [55]. Second, to address the rapid degradation and poor targeting efficiency of the bioactive substances used in regenerative medicine, researchers have developed multifunctional targeted nanoplatforms designed to mitigate these limitations. The core function of these nanocarrier systems lies in their ability to protect and efficiently deliver therapeutic agents, such as drugs [56], genes [57], and growth factors [58], to IVD lesions, while enabling controlled release [59]. This modulates the degenerative microenvironment of IVDD and promotes the restoration of cellular function. Finally, inorganic functional nanomaterials, such as metal oxide nanomaterials and carbon quantum dots, do not require additional loading of conventional drugs. By relying on their intrinsic biological activity, they can scavenge ROS [53] and regulate cellular signaling pathways [60], thereby exerting anti-inflammatory, antioxidant, and anti-aging effects at the lesion site. These innovative strategies offer broad prospects for IVDD treatment and tissue regeneration, while driving cutting-edge advances in biomedical nanotechnology. Table 1 provides a conceptual overview of the main therapeutic outcomes and key translational limitations of different nanomaterials discussed in later sections.

### 4.1. Inorganic Functional Nanozymes: Direct ROS Scavenging and Redox Regulation

In response to the pathological features of abnormal ROS accumulation and disruption of redox signaling pathways during IVDD, various nanomaterials exhibit distinct mechanisms of action. Unlike delivery-type nanomaterials, which rely on drug loading to exert antioxidant effects, inorganic functional nanomaterials can exhibit intrinsic SOD-, CAT-, or peroxidase (POD)-like enzymatic activity due to their inherent physicochemical properties. Consequently, they can directly scavenge excess ROS within cells and mitochondria without requiring additional drug loading, while also participating in the regulation of multiple signaling pathways, thereby offering unique advantages in restoring redox homeostasis within the IVD [69,70].

Cerium oxide nanoparticles (CeO_2_ NPs), a class of metal oxide nanozymes, exhibit potent catalytic activities similar to those of SOD and CAT through reversible cycling between Ce^3+^ and Ce^4+^ oxidation states on their surfaces. This catalytic property has been widely reported in nanomedicine and musculoskeletal repair contexts [71]. The pathological microenvironment of IVDD is also relevant to inflammatory-oxidative continuum, where unchecked ROS accumulation affects the function of NPCs. Consequently, leveraging the intrinsic radical-quenching capacity of these metal oxide nanozymes represents a method that can be adopted in IVDD. Wang et al. developed size-optimized PEGylated cerium oxide nanoparticles (4 nm-PEG600 CeNPs) [61]. Owing to their small size, these nanoparticles demonstrated the ability to penetrate the dense ECM structure and reach deep degenerative regions to scavenge ROS, while also showing potential to activate the PI3K/AKT pathway, thereby enhancing antioxidant defense and mitigating localized inflammatory responses during the early stages of degeneration.

Compared with metal oxide nanozymes, multi-metallic heterostructure inorganic nanozymes exhibit superior catalytic performance [70] and possess excellent optical and chemical properties [72]. Xu et al. developed a gold-silver heterostructured nanomaterial (E@Au-Ag NPs) loaded with epigallocatechin gallate (EGCG) [63]. The Au-Ag heterostructure both exhibited POD-like activity and served as a carrier for EGCG, enabling ROS scavenging at lesion site and synergistic modulation of the pathological microenvironment through EGCG delivery. In addition, the photothermal conversion properties of the Au-Ag heterostructure enable E@Au-Ag NPs to release EGCG upon near-infrared (NIR) irradiation, effectively preventing nonspecific drug release in non-target tissues while achieving precise inhibition of the NF-κB pathway and downstream pro-inflammatory mediators such as iNOS and COX-2. Although this system was evaluated in osteoarthritis rather than IVDD, the underlying therapeutic rationale of leveraging photo-responsive, dual-functional nanozymes to combat localized oxidative stress and inflammatory cascades represents a promising strategy for IVDD treatment.

Mitochondria-targeted nanozyme systems have also been explored to modulate mitochondrial reactive oxygen species (mtROS), aiming to intervene upstream in oxidative-stress amplification. Chen et al. designed a mitochondrion-targeted poly (gallic acid)-manganese nanoparticle, PGA-Mn-TP04 [62]. By utilizing the mitochondrial-targeting peptide TP04 to localize the system to NPC mitochondria and leveraging rapid electron exchange between gallic acid and manganese ions to mimic SOD-like and CAT-like activity, this platform demonstrated robust mtROS-scavenging capacity in vitro. Experimental results revealed that this intervention supported restoration of the dynamic balance between mitochondrial fusion and fission, thereby contributing to maintenance of NPC energy metabolism and potentially delaying the transition toward a catabolic phenotype.

Although inorganic functional nanozymes have demonstrated excellent enzyme-like activity and highly efficient ROS-scavenging capacity in vitro, current research has not fully clarified whether long-term in vivo retention of these materials and gradual metal ion release may pose chronic cytotoxicity and biosafety risks.

### 4.2. Bioactive Nanomaterials: Targeted Delivery of Antioxidant Regulators

While inorganic functional nanozymes directly scavenge ROS through their intrinsic enzyme-like activity, bioactive nanomaterials, represented by bio-derived vesicles and polymeric carriers, primarily achieve indirect regulation of redox homeostasis within the IVD by loading, protecting, and delivering antioxidant molecules and other bioactive substances in a targeted manner [73,74]. Although bio-derived vesicles and polymeric carriers differ in material origin and microstructure, their core design principles are to overcome physiological barriers within the IVD, enhance local accumulation efficiency of active agents, and prolong therapeutic duration. Furthermore, compared with inorganic functional nanozymes, they offer significant advantages in biosafety and biodegradability, thereby positioning them as promising delivery platforms for antioxidant therapy in IVDD [75].

#### 4.2.1. Bio-Derived Vesicles

Bio-derived vesicles primarily refer to extracellular vesicles naturally secreted by cells, including native exosomes and engineered exosomes, which possess inherently low immunogenicity and good biocompatibility [76,77]. Native exosomes do not require loading of exogenous substances, as they exert antioxidant effects through continuous release of microRNAs, proteins, and transcription factors, and other bioactive molecules [78]. For instance, mesenchymal stem cell-derived exosomes (MSC-derived EXOs) not only directly alleviate mitochondrial oxidative stress and mitigate mitochondrial dysfunction [79], but have also been shown to attenuate oxidative stress-induced ferroptosis in NPCs through regulation of the p62/KEAP1/NRF2 axis [80].

Among these, umbilical cord mesenchymal stem cell-derived exosomes (UCMSC-exos) have been reported to attenuate oxidative and inflammatory responses by targeting the NLRP3 inflammasome [81]. However, native exosomes exhibit limitations such as rapid clearance and lack of targeting specificity [78]. Consequently, engineered exosomes have emerged as a promising solution. Engineered exosomes can carry various substances with antioxidant substances [82] and can be modified for targeted delivery of antioxidant regulatory factors, thereby synergistically alleviating oxidative stress and supporting restoration of mitochondrial function.

The engineered nanocomposite CAP-sEXOs@Gel utilizes chondroitin-affinity peptides (CAP) to modify engineered exosomes, enabling targeted delivery of salvianolic acid A to EPCs [64]. In corresponding evaluation models, sEXOs entering EPCs effectively reduced intracellular iron influx and ROS production by inhibiting the HIF-2α/TfR1 pathway, thereby blocking iron-mediated ferroptosis. Furthermore, reductions in intracellular iron and ROS levels maintained mitochondrial function, reduced mtDNA leakage, and consequently inhibited activation of the cGAS-STING signaling pathway, effectively intercepting downstream inflammatory cascades in EPCs.

#### 4.2.2. Polymeric Carriers

Compared with bio-derived vesicles, polymeric carriers offer good biocompatibility and degradability, as well as advantages including high loading capacity and structural design flexibility. To address the challenge of exosome degradation in vivo, Zhang et al. designed poly(lactic-co-glycolic acid) (PLGA) microspheres [83] that encapsulate exosomes within a PLGA polymer carrier. In experimental models, this approach substantially mitigated the stability limitations of exosomes and achieved high loading efficiency, enabling sustained exosomes release. Ultimately, PLGA microspheres activated PPAR-γ expression through encapsulated MSC-derived exosomes, thereby indirectly alleviating oxidative stress in NPCs.

Furthermore, the mitochondria-targeting polymer micelles (AKG@PIDE-OPDEA) developed by Zhou et al. represent a class of functional polymer carriers that inherently combine precise delivery capability with biological activity [65]. Based on established mitochondrial-targeting strategies in nanomedicine, the amphiphilic block copolymer PIDE-OPDEA serves as the core structural component. Its hydrophilic zwitterionic OPDEA block enables low-toxicity mitochondrial targeting, thereby reducing secondary mitochondrial damage compared with traditional cationic targeting ligands. Meanwhile, the hydrophobic idebenone block, acting as a coenzyme Q10 analog, directly scavenges ROS, repairs the mitochondrial respiratory chain, and maintains mitochondrial morphology and dynamic equilibrium. Furthermore, the material’s core–shell structure enables simultaneous delivery of the TCA cycle substrate AKG to mitochondria, thereby restoring mitochondrial OXPHOS function. Through this mechanism, oxidative stress imbalance in NPCs is alleviated. Simultaneously, stabilization of mitochondrial structure reduces mtDNA, thereby effectively blocking activation of the cGAS-STING inflammatory pathway and achieving synergistic antioxidative, anti-inflammatory, and ECM metabolic repair effects.

In addition to bio-derived vesicles and polymeric carriers, liposomes are also excellent nanocarriers. Enclosed within phospholipid bilayers, liposomes can carry both hydrophilic and hydrophobic drugs, making them among the most widely used nanocarriers [84]. They can encapsulate substances such as nucleic acids and proteins, thereby shielding them from premature enzymatic degradation during transport [85,86]. However, bioactive nanomaterials also face limitations within the harsh microenvironment of degenerated IVDs. Poor intradiscal retention, off-target effects, and difficulty achieving on-demand drug release constrain their capacity to provide long-term regulation of redox homeostasis and oxidative stress [11].

### 4.3. Stimuli-Responsive Nanosystems: Smart Controlled-Release Systems for Redox Homeostasis Regulation in IVDD

Stimuli-responsive nanosystems are smart delivery platforms that utilize pathological features such as excessive ROS accumulation and acidic pH within degenerative IVDs to achieve on-demand and controlled release of therapeutic agents [87]. Unlike conventional delivery systems, these smart nanocarriers are engineered to minimize premature drug leakage and mitigate off-target accumulation, thereby enabling more precise localization of degenerative lesion sites. This strategy supports mitigation of oxidative stress-induced damage and restoration of redox balance, providing a precise and efficient approach for antioxidant therapy in IVDD.

**Figure 3:**
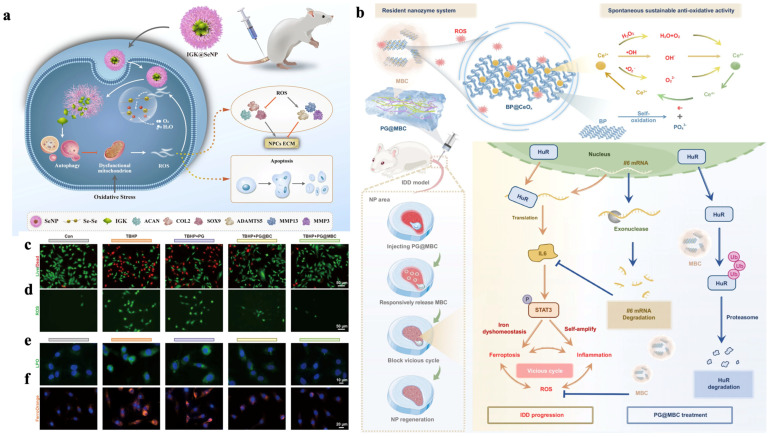
(**a**) Schematic illustration of IGK@SeNP for IVDD treatment [66]. Copyright 2023, Springer Nature. (**b**) Schematic illustration of the preparation process and disk repair mechanism of PG@MBC dynamic hydrogel. (**c**–**f**) Nanozyme-functionalized dynamic hydrogel mitigates ROS accumulation and ferroptosis in vitro [10]. Copyright 2025, Springer Nature.

ROS-responsive nanosystems are currently the most extensively studied smart delivery platforms for regulation of oxidative stress in IVDD. One example is the ROS-responsive diselenide nanoparticle IGK@SeNP loaded with isoginkgetin (IGK) [66]. This nanosystem can specifically recognizes the high-ROS microenvironment within degenerated IVDs, triggering rapid drug release upon oxidative stimulation while simultaneously exerting ROS-scavenging activity and enhancing NPCs autophagy (Figure 3).

Furthermore, multifunctional composite ROS-responsive nanosystems have been developed to further optimize controlled release and antioxidant effects. The PG@MBC system constructed by Wang et al. not only responds to the high-ROS microenvironment within degenerated IVDs to achieve controlled release of active ingredients, but also incorporates CeOx, within exerts dual SOD-like and CAT-like enzymatic activities to catalyze ROS scavenging. Furthermore, this nanosystem targets and inhibits the IL-6/STAT3 inflammatory axis, ultimately promoting IVD repair by disrupting the pathological cycle of “oxidative stress–ferroptosis–inflammation” [10] (Figure 3).

In addition to excessive ROS production, the pathological microenvironment of IVDD is also characterized by acidic pH conditions. Consequently, pH-responsive nanosystems have demonstrated excellent therapeutic potential in IVDD. The melatonin-loaded pH-responsive zeolite imidazolate framework-8 (ZIF-8) nanoparticle, MT@ZIF-8, sensitively responds to the acidic microenvironment of degenerated IVDs, enabling sustained and controlled melatonin release. Experimental results show that the melatonin released by MT@ZIF-8 effectively inhibits oxidative stress, alleviates inflammatory responses, and maintains NPC metabolic homeostasis through suppression of the NF-κB and MAPK signaling pathways [67].

Although the stimuli-responsive nanosystems demonstrated excellent performance in the aforementioned experiments by enabling controlled drug release in response to the microenvironment of degenerated IVDs, their preparation methods are more complex than those of inorganic functional nanozymes and bioactive nanomaterials, making large-scale clinical production challenging. Moreover, achieving truly controlled drug release under physiological conditions in the human body remains difficult.

### 4.4. Nanocomposite Scaffolds: Integrating Antioxidant Function with Biomechanical Restoration

Degradation of the ECM within the NP, particularly the loss of aggrecan and type II collagen, significantly impairs NPC function. The application of nanotechnology in ECM reconstruction has evolved from merely providing only mechanical support to developing nanocomposite scaffolds that mimic ECM properties and nanocarriers capable of precisely delivering matrix components [88,89]. These scaffolds not only overcome several limitations of traditional scaffolds but also integrate antioxidant properties, biomimetic ECM structure, and biomechanical adaptability. While providing structural support for degenerated IVDs, these scaffolds scavenge ROS and regulate redox homeostasis, thereby achieving simultaneous histological and biomechanical repair.

Nanocomposite hydrogels are the most representative class of nanocomposite scaffolds. They exhibit good biocompatibility and mimic the structure and properties of the native ECM, thereby providing a supportive microenvironment conducive to cellular survival and repair within the IVD [55]. In addition, they allow incorporation of anti-inflammatory and antioxidant agents into the hydrogel matrix, simultaneously modulating the IVDD microenvironment [90]. Compared with nanocomposite hydrogels, traditional hydrogels exhibit limitations such as inadequate mechanical properties [91], limited biocompatibility [92], poor stability [93], and a lack of environmental responsiveness [94], making it difficult for them to function effectively within the complex pathological environment of the IVD. In contrast, nanocomposite hydrogels are designed to largely overcome these shortcomings and perform multiple functions within the degenerated IVD environment.

**Figure 4:**
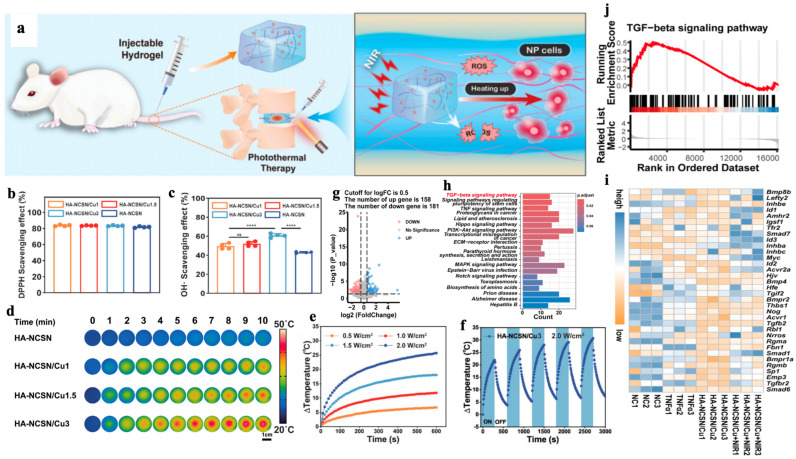
(**a**) Restoration of IVDD by the HA-NCSN/Cu hydrogel through antioxidant and photothermal therapy. (**b**) DPPH radical-scavenging activity of HA-NCSN/Cu hydrogels. (**c**) Hydroxyl radical scavenging ability of HA-NCSN/Cu hydrogels. (**d**) Thermal images of hydrogels with different Cu^2+^ concentrations after 10 min of NIR irradiation. (**e**) Photothermal heating curves of the HA-NCSN/Cu3 hydrogels at different NIR power densities. (**f**) Photothermal curves of the HA-NCSN/Cu3 hydrogel during five cycles of NIR irradiation at a power density of 2.0 W cm^−2^. (**g**) Volcano plot. (**h**) KEGG enrichment analysis. (**i**) Heatmap. (**j**) GSEA analysis. [68] Copyright 2024, Wiley-VCH. Significance levels are as follows: **** *p* < 0.0001.

For instance, Bu et al. [68] designed an HA-NCSN/Cu hydrogel that directly regulates its crosslinking density and mechanical strength by adjusting the concentration of the crosslinking agent copper ions (Cu^2+^), thereby mimicking the elastic modulus of native NPs (Figure 4). Furthermore, the hyaluronic acid (HA) backbone of the HA-NCSN/Cu hydrogel provides structural support typical of conventional hydrogels. The grafted thiourea groups were shown to efficiently scavenge accumulated ROS, thereby alleviating inflammatory and oxidative stress responses in NPCs and demonstrating responsive properties in experimental models. Notably, Cu^2+^ within the HA-NCSN/Cu hydrogel can also serve as a catalyst for photothermal therapy through a reduction-induced color-change reaction, thereby activating the TGF-β/Smad signaling pathway. In evaluated models, this cascade upregulated the expression and secretion of aggrecan and collagen II while downregulating MMP3 and MMP13 expression, promoting ECM reconstruction.

In addition, gelatin, which shares structural similarities with the ECM, is commonly used in the synthesis of nanocomposite hydrogels to promote cell proliferation and ECM deposition [92]. In particular, the immunoprotective gelatin microspheres (NM-PLGA@TGF-β1-GMs) developed by Zhou’s team not only mimic the physical properties of the ECM but also clear free inflammatory factors from the degenerative microenvironment through neutrophil membranes encapsulated on the surface of the PLGA particles. Together, these mechanisms provide a stable microenvironment with antioxidant and anti-inflammatory properties that support NPC repair [95].

Nanocomposite scaffolds can synergistically regulate IVDD by providing both mechanical support and antioxidant effects. However, in the aforementioned studies, these nanomaterials were primarily administered via local injection, which may cause secondary damage to the IVD and compromise AF integrity. Additionally, long-term biomechanical durability remains an important consideration for clinical translation.

## 5. Challenges of Nanomaterials in IVDD Treatment

Although nanomaterials have demonstrated antioxidant, anti-inflammatory, and anti-degenerative potential in preclinical studies of IVDD, all positive findings to date have been derived solely from proof-of-concept animal studies, which fundamentally differ from clinically realistic therapeutic strategies. Consequently, nanomaterials continue to face substantial barriers in the transition from laboratory research to practical clinical application.

### 5.1. Process Challenges in Large-Scale Production and Quality Control

Nanomaterials at the proof-of-concept stage are typically produced in small laboratory batches, whereas clinical applications require standardized, scalable, and reproducible industrial manufacturing. Furthermore, as the designs of nanomaterials become increasingly sophisticated and multifunctional, the complexity of chemical synthesis and quality control continues to increase, thereby becoming a major bottleneck for large-scale industrial production [96].

The preparation of multifunctional nanomaterials, such as engineered exosomes and composite nanohydrogels, involves complex procedures including biomaterial extraction, surface modification, and biomimetic assembly. Variability between synthesis batches may occur, and for nanomaterials such as extracellular vesicles, unified industrial standards defining purity, potency, and storage stability are still lacking [97,98], making it difficult to satisfy good manufacturing practice requirements.

Simultaneously, material sterilization and storage stability must also be considered in clinical applications. Conventional sterilization methods, such as gamma irradiation, high-pressure steam sterilization, and filtration sterilization, can damage structures of nanomaterials and impair functionality, whereas laboratory-based aseptic synthesis methods ae difficult to adapt to industrial production conditions [99]. Furthermore, the long-term storage stability of most nanomaterials remains poorly characterized. Under low-temperature or ambient conditions, these materials are prone to agglomeration, drug leakage, and ligand inactivation, rendering them unsuitable for clinical storage and transport requirements [100]. Although these issues are often overlooked in proof-of-concept animal studies, they represent substantial technical barriers to clinical translation.

### 5.2. Effectiveness Within the Actual Pathological Environment of IVD Remains Uncertain

Under normal physiological conditions, the IVD is subjected to immense axial pressure. Following degeneration, it develops into a harsh acidic environment characterized by hypoxia and high levels of ROS, which poses a challenge to the stability of nanomaterial stability. Due to the enclosed high-pressure anatomical characteristics of the IVD, conventional free-form nanoparticles are highly susceptible to leakage through fissures in the degenerated AF or CEP during repetitive spinal compression, resulting in rapid decline of local drug concentrations.

Concurrently, elevated ROS levels and acidic conditions not only accelerate nonspecific degradation of nanocarriers but may also inactivate sensitive ligands, thereby shortening the therapeutic window [54]. Currently, most proof-of-concept animal studies employ acute degeneration models with observation periods of only 2–4 weeks, which fail to adequately simulate the pathological microenvironment of human IVDD, including chronic degeneration, sustained axial compression, hypoxia, elevated ROS levels, and pathological acidic pH [101,102]. For clinical treatment, nanomaterials must remain functional within the avascular high-pressure IVD microenvironment for months to years. Consequently, the aforementioned limitations may hinder the long-term therapeutic performance of nanomaterials within the pathological IVD environment. The short-term functional stability observed in proof-of-concept studies does not necessarily translate into durable clinical efficacy.

### 5.3. Potential Risks Related to Long-Term Biosafety and Immunogenicity

IVDD is a chronic degenerative disease that often requires prolonged therapeutic retention within the body to maintain efficacy [78]. Consequently, nanomaterials may pose risks of chronic accumulation, toxicity, and immune reactions in vivo. Inorganic nanomaterials synthesized from metals such as gold, silver, and manganese exhibit excellent photothermal and catalytic properties; however, their metabolic clearance pathways remain insufficiently understood [103,104]. Although short-term studies have not revealed significant organ toxicity associated with these nanomaterials, long-term accumulation of metal ions within the enclosed IVD microenvironment or distant organs such as the liver and kidneys still pose risks of heavy metal toxicity.

Regarding nanomaterials involving gene-reprogramming strategies, such as OKS@M-Exo delivering Oct4/Klf4/Sox2 plasmids, current studies have shown no evidence of tumor-promoting effects. Nevertheless, long-term clinical application of induced pluripotency factors still requires careful monitoring for teratoma formation and off-target effects [105]. Furthermore, materials such as TMNP@SR that utilize macrophage membranes for immune evasion have demonstrated short-term immune escape capability. However, long-term primate studies are still required to determine whether heterologous proteins or modified surface receptors may induce acquired immune responses during prolonged administration, thereby reducing therapeutic efficacy or causing allergic reactions. These safety concerns cannot be excluded based on short-term animal studies and remain inconsistent with the long-term biosafety requirements of clinical translation.

### 5.4. Method of Administration Carries a Risk of Exacerbating Disk Damage

Currently, drug administration in animal studies is primarily achieved through intradiscal injections. Although local injection can overcome the physiological barrier of the IVD and deliver high concentrations of drugs into the NP, this invasive approach can cause secondary damage to degenerated IVDs [106]. Experimental observations indicate that needle-based drug delivery, while enabling efficient drug administration, also causes damage to the AF. As a critical barrier structure of the IVD, AF damage triggers a cascade of pathological changes, including the sustained release of inflammatory mediators, such as IL-6 and MMP13, as well as oxidative damage markers, such as 8-OHdG, further exacerbating the disruption of the structural and functional stability of the IVD [107].

Meanwhile, some studies have employed systemic administration methods such as intravenous or intraperitoneal injection, which are less invasive than local injection. However, following administration, nanomaterials are rapidly cleared by organs such as the liver and spleen, which significantly reduces drug delivery efficiency. Furthermore, the physiological characteristics of the IVD, including its avascular nature, and the physical properties of proteoglycans within the NP matrix, which possesses a net negative charge, impose stringent requirements on nanoparticle size and surface charge.

To address these ongoing challenges, researchers are actively exploring strategies various preclinical strategies. For instance, Panebianco et al. developed a dual-network composite hydrogel scaffold that utilizes a high-modulus, genipin-crosslinked fibrinogen outer matrix to achieve immediate sealing of AF defects following localized injection [108]. This platform not only reconstructed the internal biomechanical barrier of the IVD to prevent further NP tissue herniation in vitro, but also served as a protective matrix that enhanced the retention of exogenously seeded cells within the lesion, preventing their leakage and dissipation in a hyper-pressurized microenvironment. Similarly, Wang et al. utilized nanostructured gelatin colloidal gels with shear-thinning and self-healing properties to enable low-resistance, minimally invasive injection through a 21G needle, thereby significantly reducing mechanical damage to the AF in animal models. Upon injection, the hydrogel recovered its solid-like state in situ, forming an effective leak-proof barrier that prevented NP leakage while markedly improving the survival and retention of encapsulated cells [109]. From a translational perspective, clinicians currently employ ultrasound-guided intradiscal injections for conventional therapeutic delivery, providing symptom relief without significant complications [109]. However, the adaptation of this delivery route for advanced nanomaterials remains at an early stage. Although these injectable, biomimetic nanomaterials offer a promising platform for targeted delivery, extensive preclinical validation is still required before they can be safely translated into clinical practice.

Furthermore, existing studies lack standardized dosing systems. Dosages in animal experiments are often selected empirically without establishing dose-conversion standards that account for human IVD volume, mechanical loading, and degeneration severity, thereby limiting their applicability for precise clinical dose optimization.

### 5.5. Regulatory Barriers and the Lack of Long-Term Efficacy Monitoring

Currently, uniform global regulatory guidelines for nanomaterials are lacking. Composite formulations comprising nanocarriers, bioactive payloads, and cell-derived components also lack standardized criteria for evaluating purity, potency, safety, and stability, resulting in insufficient regulatory frameworks for clinical application [110]. First, for inorganic functional nanozymes, current research has not yet established whether these materials are associated with long-term toxicity or foreign body reactions. Consequently, regulatory agencies require extensive long-term safety data, which remain insufficient for most inorganic functional nanozymes [11]. Second, bioactive nanomaterials lack standardized isolation and purification protocols. In addition, the establishment of uniform quality control standards remains challenging because of the technical limitations of existing quantification assays [111]. Finally, for nanocarrier-based systems, fabrication processes are often complex, making compliance with GMP standards and the achievement of consistent, scalable, batch-to-batch production difficult. Data from proof-of-concept animal studies can only validate the theoretical feasibility of these nanomaterials and fail to satisfy the core requirements of regulatory authorities regarding long-term safety, large-scale manufacturing, and quality control. Furthermore, clinical translation must be accompanied by systems for long-term efficacy and safety monitoring; however, existing studies have focused primarily on short-term histological and imaging changes in animal models. Long-term follow-up protocols for monitoring nanomaterials persistence in vivo, sustained payload release, organ toxicity, and recurrence of degeneration remain underdeveloped, making comprehensive evaluation of long-term benefits and risks difficult.

In summary, current research on nanotherapies for IVDD remains at the animal proof-of-concept stage and primarily demonstrates theoretical feasibility. Considerable challenges remain before clinically viable strategies suitable for scalable manufacturing, regulatory compliance, long-term safety, controllability, minimally invasive delivery, and durable therapeutic efficacy can be developed.

Future research must move beyond the limitations of short-term animal experiments and focus on clinical requirements such as scalable manufacturing, sterilization stability, long-term biosafety, minimally invasive delivery, and regulatory compliance to realize the translational application of nanomaterials in IVDD therapy.

## 6. Summary and Outlook

Over the past decade, owing to their unique physicochemical properties and functional modifiability, nanomaterials have been investigated as tools to address some biological barriers of the IVD, particularly by improving local retention, controlled release, and microenvironment-responsive delivery. These platforms may help address several limitations of conventional treatments and are promising tools for modulating oxidative stress and redox imbalance in IVDD.

In the context of the challenges faced by IVDs as the disease progresses, nanomaterials can exert therapeutic effects through various antioxidant mechanisms. Antioxidant nanozymes can directly scavenge excess ROS through enzyme-like activity. Bioactive nanomaterials can deliver redox modulators, and stimuli-responsive nanosystems can exploit the pathological characteristics of excess ROS and pathological pH in degenerated IVDs to achieve controlled release of therapeutic drugs. Moreover, nanocomposite scaffolds can integrate antioxidant capacity with biomechanical support. In preclinical models, these strategies have been reported to restore redox balance, protect NPCs and EPCs from oxidative damage, inhibit inflammation and cell death induced by oxidative stress, and mitigate ECM degradation, thereby exerting further beneficial effects.

Current research has also shifted from the development of nanomaterials targeting a single pathway toward the development of multifunctional composite nanomaterials. Although these materials have demonstrated considerable potential in animal studies, significant challenges remain before nanotherapy can be translated into clinical practice.

**Figure 5:**
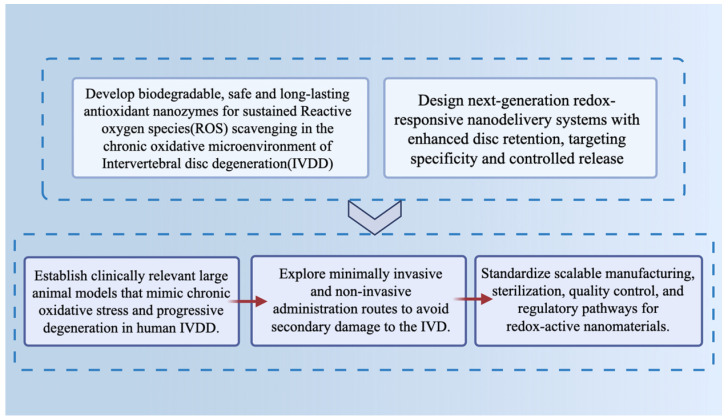
Proposed developmental and translational roadmap for redox-modulating nanotherapeutics in IVDD treatment. Created in BioRender. Yingzi Zhou. (2026). https://BioRender.com/n75ytg4 (accessed on 13 May 2026).

In addition to designing multifunctional nanomaterials based on the pathological mechanisms of oxidative stress and redox imbalance in the IVDD, researchers should actively develop clinically relevant large-animal models capable of realistically simulating chronic oxidative stress, sustained mechanical loading, and progressive pathological degeneration observed in human IVDD. Simultaneously, less invasive and image-guided delivery methods, together with annulus-preserving or annulus-sealing strategies, should be prioritized to minimize secondary IVD injury during repeated or long-term treatment. Furthermore, standardized and scalable production processes, sterilization procedures, quality control systems, and regulatory pathways for redox-active nanomaterials must be established. To synthesize these future trajectories, Figure 5 provides a conceptual overview of the proposed developmental and translational roadmap for redox-modulating nanotherapeutics in IVDD treatment.

Comprehensively addressing these challenges is essential for advancing nanotechnology from laboratory proof-of-concept to clinically viable therapeutic approaches, ultimately enabling effective correction of redox imbalance within the IVD and achieving structural repair and functional regeneration of degenerated IVDs.

## Figures and Tables

**Table 1:** Main outcomes and key translational limitations of different nanomaterials.

Study	Formulation	Experimental Model and Target	Administration Method	Experiment Duration	Main Outcomes	Key Translational Limitations
4 nm-PEG600-CeNPs [61]	Metal oxide nanomaterial	Rat IVDD model NPCs	In situ injection (IS)	8 w	Modulated ROS accumulation triggered NP cell senescence.	High cytotoxicity and inadequate tissue retention.
PGA-Mn-TP04 [62]	Mitochondria-targeted nanozyme	Rat IVDD model Mitochondria of NPCs	IS	8 w	Scavenged mtROS and restored mitochondrial function to alleviate oxidative stress in NPCs.	Unknown long-term metabolism and potential chronic accumulation of manganese ions in the avascular disk.
E@Au-Ag NPs + EGCG [63]	Multi-metallic heterostructure inorganic nanozyme	Rat osteoarthritis model Mitochondria of Chondrocytes	IS	8 w	Scavenged ROS and protected chondrocyte mitochondria.	(1) Unknown long-term degradation kinetics and clearance pathways of Au/Ag noble metals in joints. (2) Limited tissue penetration depth of external NIR laser through thick human periarticular tissue. (3) Clinical risk of articular structural damage and retrograde joint infection caused by recurrent intra-articular injections.
CAP-sEXOs@Gel [64]	Engineered exosomes	Rat IVDD model EPCs and NPCs	IS	8 w	(1) Scavenged intracellular ROS to mitigate iron-induced oxidative stress and promote EPC survival. (2) Suppressed EPC inflammatory secretion to indirectly rescue NPCs from oxidative senescence.	(1) Intricate multi-step preparation and difficult quality control. (2) Unoptimized therapeutic dosage and administration frequency. (3) Biomechanical and etiological mismatches in animal models.
AKG@PIDE-OPDEA [65]	Polymer micelles	Rat IVDD model Mitochondria of NPCs	IS	12 w	(1) Targeted mitochondria precisely to maintain homeostasis and restore energy metabolism. (2) Prevented mitochondrial DNA leakage to block cGAS-STING activation, reducing injury and inflammation.	(1) Unknown long-term degradation kinetics in avascular disk. (2) Translational dilemma between micelle retention and recurrent punctures.
IGK@SeNP [66]	ROS-responsive nanosystem	Rat IVDD model NPCs	IS	8 w	Synergistically eliminated ROS and enhanced autophagy to protect NPCs.	Lack of in vivo hemocompatibility and long-term systemic toxicity evaluations.
MT@ZIF-8 [67]	pH-responsive nanosystem	Rat IVDD model NPCs	IS	4 w	Scavenged ROS and suppressed inflammation to protect NPCs.	(1) Unclear pharmacokinetics and optimal in vivo dosage. (2) High technical dependence on precise minimally invasive puncture to prevent secondary spinal nerve root trauma.
HA-NCSN/Cu hydrogel [68]	Nanocomposite hydrogel	Rat IVDD model NPCs	IS	8 w	Scavenged ROS and suppressed inflammation to protect NPCs by regulating glutathione metabolism.	(1) Limited tissue penetration depth of external NIR laser in humans. (2) Potential risk of long-term copper ion accumulation and cuproptosis in avascular disk.

## Data Availability

No new data were created or analyzed in this study. Data sharing is not applicable to this article.

## Abbreviations

The following abbreviations are used in this manuscript:

ADAMTS	A disintegrin and metalloproteinase with thrombospondin motifs
AF	Annulus fibrosus
AKG	α-Ketoglutaric acid
ARE	Antioxidant response element
C. acnes	Cutibacterium acnes
CAP	Cartilage-affinity peptide
CAT	Catalase
CEP	Cartilaginous endplate
cGAS	Cyclic GMP-AMP synthase
DAMPs	Damage-associated molecular patterns
ECM	Extracellular matrix
EGCG	Epigallocatechin gallate
EPCs	Endplate chondrocytes
HA	Hyaluronic acid
HIF-2α	Hypoxia-inducible factor-2 alpha
IGK	Isoginkgetin
IL-1β	Interleukin-1beta
IL-6	Interleukin-6
IVD	Intervertebral disk
IVDD	Intervertebral disk degeneration
Keap1	Kelch-like ECH-associated protein 1
LBP	Low back pain
Maf	Musculoaponeurotic fibrosarcoma
MAPK	Mitogen-activated protein kinase
MMPs	Matrix metalloproteinases
MSC-derived EXOs	Mesenchymal stem cell-derived exosomes
mtROS	Mitochondrial reactive oxygen species
NF-κB	Nuclear factor kappa-B
NIR	Near-infrared
NLRP3	NLR family pyrin domain containing 3
NP	Nucleus pulposus
NPCs	Nucleus pulposus cells
Nrf2	Nuclear factor erythroid 2-related factor 2
OXPHOS	Oxidative phosphorylation
PIDE-OPDEA	Poly(idebenone)-poly (2-(N-oxide-N, N-dimethylamino) ethyl methacrylate)
PLGA	Poly (lactic-co-glycolic acid)
POD	Peroxidase
PPAR-γ	Peroxisome proliferator-activated receptor gamma
ROS	Reactive oxygen species
SASP	Senescence-associated secretory phenotype
SOD	Superoxide dismutase
STING	Stimulator of interferon genes
TCA	Tricarboxylic acid cycle
TfR1	Transferrin receptor 1
TLR	Toll-like receptor
TNF-α	Tumor necrosis factor-alpha
TXNIP	Thioredoxin-interacting protein
UCMSC-Exos	Umbilical cord mesenchymal stem cell-derived exosomes
ZIF-8	Zeolite imidazolate framework-8
